# Cost-effectiveness and social outcomes of a community-based treatment for podoconiosis lymphoedema in the East Gojjam zone, Ethiopia

**DOI:** 10.1371/journal.pntd.0007780

**Published:** 2019-10-23

**Authors:** Natalia Hounsome, Meseret Molla Kassahun, Moses Ngari, James A. Berkley, Esther Kivaya, Patricia Njuguna, Greg Fegan, Abreham Tamiru, Abebe Kelemework, Tsige Amberbir, Annabelle Clarke, Trudie Lang, Melanie J. Newport, Andy McKay, Fikre Enquoselassie, Gail Davey

**Affiliations:** 1 Brighton and Sussex Centre for Global Health Research, Brighton and Sussex Medical School, University of Sussex, Brighton, United Kingdom; 2 Department of RH and Health Services, School of Public Health, College of Health Sciences, Addis Ababa University, Addis Ababa, Ethiopia; 3 KEMRI/Wellcome Trust Research Programme, Kilifi, Kenya; 4 Centre for Tropical Medicine and Global Health, University of Oxford, Oxford, United Kingdom; 5 Swansea University Medical School, Swansea, United Kingdom; 6 International Orthodox Christian Charities Podoconiosis Project, Debre Markos, Ethiopia; 7 Department of Economics, University of Sussex, Brighton, United Kingdom; 8 School of Public Health, Addis Ababa University, Addis Ababa, Ethiopia; RTI International, UNITED STATES

## Abstract

**Background:**

Podoconiosis is a disease of the lymphatic vessels of the lower extremities that is caused by chronic exposure to irritant soils. It results in leg swelling, commonly complicated by acute dermatolymphangioadenitis (ADLA), characterised by severe pain, fever and disability.

**Methods:**

We conducted cost-effectiveness and social outcome analyses of a pragmatic, randomised controlled trial of a hygiene and foot-care intervention for people with podoconiosis in the East Gojjam zone of northern Ethiopia. Participants were allocated to the immediate intervention group or the delayed intervention group (control). The 12-month intervention included training in foot hygiene, skin care, bandaging, exercises, and use of socks and shoes, and was supported by lay community assistants. The cost-effectiveness analysis was conducted using the cost of productivity loss due to acute dermatolymphangioadenitis. Household costs were not included. Health outcomes in the cost-effectiveness analysis were: the incidence of ADLA episodes, health-related quality of life captured using the Dermatology Life Quality Index (DLQI), and disability scores measured using the WHO Disability Assessment Schedule 2.0 (WHODAS 2.0).

**Results:**

The cost of the foot hygiene and lymphoedema management supplies was 529 ETB (69 I$, international dollars) per person per year. The cost of delivery of the intervention as part of the trial, including transportation, storage, training of lay community assistants and administering the intervention was 1,890 ETB (246 I$) per person. The intervention was effective in reducing the incidence of acute dermatolymphangioadenitis episodes and improving DLQI scores, while there were no significant improvements in the disability scores measured using WHODAS 2.0. In 75% of estimations, the intervention was less costly than the control. This was due to improved work productivity. Subgroup analyses based on income group showed that the intervention was cost-effective (both less costly and more effective) in reducing the number of acute dermatolymphangioadenitis episodes and improving health-related quality of life in families with monthly income <1,000 ETB (130 I$). For the subgroup with family income ≥1,000 ETB, the intervention was more effective but more costly than the control.

**Conclusions:**

Whilst there is evident benefit of the intervention for all, the economic impact would be greatest for the poorest.

## Author summary

Podoconiosis is a disease of the lymphatic vessels of the lower extremities that is caused by chronic exposure to irritant soils. The condition has been reported from 32 countries around the world, and is common in low-resource settings in which people do not wear shoes as protection against the soil. It results in leg swelling, commonly complicated by acute dermatolymphangioadenitis (ADLA, or ‘acute attacks’), characterised by severe pain, fever and disability. A low-cost, lay-led treatment package to prevent ADLA and reverse early stages of disease was tested in northern Ethiopia in 2014–2015. The package involves daily foot hygiene, skin care elastic bandaging where necessary, exercises, elevation and regular use of socks and shoes. In this study we report the impact of this package on quality of life and decreasing work productivity losses.

## Introduction

Podoconiosis is a form of leg swelling due to lymphoedema arising from exposure to red clay soils derived from alkalic volcanic rock [1]. It is most prevalent in extremely poor subsistence farming communities who spend the majority of their time working barefoot in irritant soils. Podoconiosis results in debilitating mobility issues, affecting persons' earning ability and quality of life, in particular, due to acute dermatolymphangioadenitis episodes (ADLA) characterised by severe pain, fever and disability.

A recent systematic review indicates that podoconiosis has been reported at some point in time from 32 countries worldwide, 18 in the African Region, 3 in Asia and 11 in Latin America [2]. The highest reported prevalence values were in Africa (8.08% in Cameroon, 7.45% in Ethiopia, 4.52% in Uganda, 3.87% in Kenya and 2.51% in Tanzania).

Podoconiosis leads to significant stigma and reduced quality of life [3–5]. It has significant impact on productivity, with affected people losing 45% of total working days per year [6]. However, misdiagnosis (chiefly confusion with filarial lymphoedema) and fatalism have hampered treatment [7]. Only in the past two decades has foot hygiene treatment been offered specifically to people with podoconiosis [8,9], and this has been initiated by non-government organisations without state adoption or systematic evaluation. Based on the urgent need for evidence on which to base national policy, a fully randomised controlled trial (RCT) of the intervention was conducted in northern Ethiopia [10].

The Gojjam Lymphoedema Best Practice Trial (GoLBeT) was a pragmatic RCT designed to evaluate the effectiveness of podoconiosis lymphoedema management in the community (ISRCTN67805210). The RCT set out to test the hypothesis that community-based treatment of podoconiosis lymphoedema reduces the frequency of acute dermatolymphangioadenitis episodes and secondarily that it improves other clinical, social and economic outcomes. Podoconiosis patients aged 18 years and older were individually randomised to a package comprising instruction in foot hygiene, skin care, bandaging, exercises, use of socks and shoes, with support by lay Community Podoconiosis Agents (CPAs) at monthly intervention meetings; or to no intervention. Full details of the RCT protocol and the Rapid Ethical Assessment that preceded recruitment are described in previous articles [10, 11]. The primary outcome results have been published [12], as has detailed description of the screening and enrolment process [13].

Although several economic analyses of lymphatic filariasis (LF) treatment exist and demonstrate high cost-effectiveness [14], there are no previous estimates of the cost-effectiveness of podoconiosis treatment. Here we present the results of a cost-effectiveness analysis of the GoLBeT trial, with scenario analysis to reflect the approach to provision of care promoted by the Ethiopian Federal Ministry of Health [15].

## Methods

### Ethics statement

The study was approved by the Institutional Review Board of the College of Health Sciences, Addis Ababa University (071/13/SPH), the National Ethical Review Committee of the Ethiopian Science and Technology Agency (3-1/794/06), the Food, Medicine and Health Care Administration Authority of Ethiopia (02/6-1/05/39), and the Research Ethics and Governance Committee of Brighton and Sussex Medical School (13/107/DAV).

### Data sources and study characteristics

Study design, setting and participants' characteristics are described in detail elsewhere [10]. Briefly, participants were adults aged 18 years and older, with a diagnosis of at least stage 2 podoconiosis (persistent lymphoedema). Participants were individually randomised (1:1 ratio) to the intervention or control groups. Participants randomised to the intervention group were instructed in foot hygiene, skin care, bandaging, exercises, and use of socks and shoes, and supported by lay community assistants at monthly meetings. Control patients were followed up quarterly for data collection, but received no intervention for 12 months (delayed intervention). The primary outcome was incidence of ADLA, measured using a validated patient-held pictorial diary. Secondary outcomes included: quality of life measure using the Amharic version of the Dermatology Life Quality Index II (DLQI) [16, 17]; disability scores measured using the 12-item version of the WHO Disability Assessment Schedule 2.0 (WHODAS 2.0) [18]; days totally unable to work and days with reduced productivity; stigma; and observed clinical changes. The number of ADLA episodes and DLQI questionnaires were collected at baseline, 3, 6, 9 and 12 months; family income data were collected at baseline, 6 and 12 months. WHODAS 2.0 questionnaires, days totally unable to work and days with reduced productivity were collected at baseline and 12 months.

### Health economics analysis

The economic evaluation was conducted from a mixed perspective including a programme implementation perspective and a patient perspective. Study participants were predominantly unpaid subsistence farmers who produce food for their own consumption. Therefore, we analysed work productivity from a patient perspective (direct costs). Economic evaluation methods followed the WHO guide to cost-effectiveness analysis [19], which includes recommendations on estimating costs, health outcomes and assessing uncertainty in cost-effectiveness analysis.

### Costing GoLBet intervention

The economic cost of the intervention was estimated using financial records and interviews with project managers. Economic analysis was not anticipated in this study. Therefore, data on resource use and associated costs were collected retrospectively. We used a micro-costing approach involving bottom-up construction of costs associated with setting up and delivering the intervention. The costing categories included: treatment supplies, storage and transportation; community assistants’ salaries; training community assistants; community podoconiosis activities; education and awareness raising and administration costs. We excluded research costs associated with trial set-up and data collection (e.g. research staff, computers, statistical software, medical and life insurance, ethics approval and audit). The estimated cost of the intervention per person was based on the number of participants enrolled in the trial (intention to treat). The average cost per participant was estimated with and without training to reflect the fact that, once trained, the community assistants might continue to fulfil their duties beyond the duration of the trial. The summary of intervention costs is shown in Table 1.

**Table 1:** Summary of set-up and operational costs of community-based treatment of podoconiosis lymphoedema.

**Hygiene and treatment supplies**	**Units**	**Duration**	**Resource use**	**Unit cost, ETB**	**Total cost,** **ETB**
Ointment	1 tube	12 months	100%	10	83,520
Soap	1 piece	12 months	100%	8	66,816
Shoes	1 pair	once	99%	270	186,300
Socks	2 pairs	once	100%	19	13,224
Basin	1 item	once	100%	25	17,400
Bandages	1 pack	once	5%	20	696
**Sub-total (hygiene and treatment supplies)**					**367,956**
**Per patient (n = 696)**					**529**
**Intervention delivery **	**Units**	**Duration**	**Resource use**	**Unit cost, ETB**	**Total cost,** **ETB**
**Salaries**					
Community Assistants	15	12 months		950	171,000
Administrator	1	12 months		14,000	168,000
**Storage of supplies**		36 months		lump sum sum	25,173
**Transportation**					
Car rental		36 months		lump sum	703,687
Fuel		36 months		lump sum	141,587
**Training community assistants**	18 assistants	4 days initial training + 3 days refresher	2 trainers	lump sum	27,600
**Monthly meetings with patients** (18 *kebele*)	2–18 people	3 hours	2–3 assistants	lump sum	55,000
**Community education & awareness activities**					
Officials Sensitization Workshop	46 officials	3 hours	3 trainers		
Community Sensitization Workshops(4 kebele)	12–30 people	2 hours	2 trainers		
**Total (education & awareness)**				lump sum	23,595
**Sub-total (delivery)**					**1,315,638**
**Per patient (n = 696)**					**1,890**
**Total cost of intervention**					**1,683,594**
**Cost per patient including training**					**2,419**
**Cost per patient excluding training**					**2,379**

Numbers are rounded to nearest ETB (Ethiopian Birr)

### Health-related productivity loss

The loss of productivity due to podoconiosis illness was estimated by summing the number of days unable to work and days with reduced activity. It was assumed that days with reduced activity account for a 50% reduction in work productivity. This assumption was based on a study which estimated that the cost of work presenteeism (all causes of illness) amounts to approximately half of the cost of sickness absence [20].

The average number of days with lost productivity over 12 months was estimated using the area-under-the curve method (trapezoidal) [21]. Productivity loss was costed using the average daily wages (poor persons’ general consumer price index-deflated real wages) for unskilled rural labour in Ethiopia [22]. This study was based on data on prevailing wages collected by Central Statistical Agency of Ethiopia (2015). We used average wages for Amhara region in 2015 (37.1 ETB) [22, page 27]. All costs were converted to I$ and adjusted to the year 2016 using the Purchasing Power Parity converter [23] to alleviate currency exchange rate fluctuations.

### Effectiveness outcomes

The health economics analysis was conducted using three effectiveness outcomes: the number of ADLA episodes, DLQI and WHODAS 2.0.

DLQI [16] is a dermatology-specific Quality of Life instrument, which was translated into Amharic and validated among patients with podoconiosis in southern Ethiopia [17]. It consists of 10 questions concerning patients’ perception of the impact of skin diseases on different aspects of their life, including symptoms and feelings, daily activities, leisure, work or school and personal relationships. Each question is scored on a 4-point Likert scale (0–3). The scores of individual items are added together to yield a total score ranging from 0 to 30. Higher scores mean lower quality of life [16].

WHODAS 2.0 is the World Health Organization Disability Assessment Schedule, a generic instrument for assessing health and disability [18]. It includes 12 items covering cognition, mobility, self-care, interaction with people, life and community activities. The Amharic version of WHODAS 2.0 was validated in people with severe mental disorders in rural Ethiopia [24]. The items are rated using a 5-point Likert scale (1–5). Higher scores mean greater disability. DLQI and WHODAS 2.0 total scores were calculated by adding the scores for each item. To enable the cost-effectiveness analysis, the number of ADLA episodes, DLQI and WHODAS 2.0 for each participant were calculated over 12 months using the area-under-the curve method (trapezoidal) [21].

### Missing data

Multiple imputations [25, 26] were carried out using the predictive mean matching method [27] in Stata 12.1. Missing data were assumed to be missing at random. For each missing category, five datasets were imputed. For DLQI and WHODAS 2.0 the answers to missing questions were imputed based on the following covariates: age, gender, randomisation group and *kebele* (smallest administrative unit in Ethiopia). Missing data for days totally unable to work and days with reduced activity were imputed based on the number of ADLA episodes, age, gender, randomisation group and *kebele*. Missing data were not imputed for family income or the number of ADLA episodes.

### Cost-effectiveness analysis

The cost-effectiveness analysis was conducted on an intention-to-treat basis. We assessed the incremental changes in costs associated with productivity loss due to illness and the incremental changes in the effectiveness outcomes: the number of ADLA episodes, DLQI and WHODAS 2.0. Both costs and effectiveness outcomes were adjusted for covariates age, gender, and *kebele* using three linear models: generalised linear model (GLM), seemingly unrelated regression (SUR) and multilevel mixed-effect model (MLM). The GLM model assumed gamma distribution and identity link. The SUR model included regression equations for both cost and effectiveness outcomes, each regressed on the age, gender, and *kebele*. The MLM model included age and gender as a fixed effect and *kebele* as a random effect. The incremental cost-effectiveness ratio was calculated as a difference in cost between the immediate and delayed groups divided by the difference in effectiveness outcome between the immediate and delayed groups. A non-parametric bootstrap method was used to assess the uncertainty in cost-effectiveness ratios. Results of cost-effectiveness analyses are presented as cost-effectiveness planes (incremental cost plotted against the incremental outcome). Subgroup analyses were conducted for individuals with two different levels of family income: lower (<1,000 ETB month) and higher (≥1,000 ETB/month), this cut-off being based on a baseline economic survey conducted in a neighbouring district prior to the trial [28]. It should be mentioned that although we divided participants into “lower” and “higher” income groups, they are all members of a very poor population who often cannot afford shoes, socks, or even water to wash their feet. Scenario analysis considered the cost of the intervention without training community assistants, assuming that once trained they will continue fulfilling their duties.

## Results

### Intervention costs

Costs associated with set-up and delivery of community-based treatment for podoconiosis lymphoedema are summarised in Table 1. These were divided into the costs of treatment supplies and the costs of delivering the intervention. The treatment supplies included: Whitfield ointment (one tube per month); soap (one piece per month); custom-made shoes (one pair); socks (two pairs); bandages (one pair) and basin (one). Whitfield ointment and soap were provided over 12 months. Bandages were required for 5% of patients only. The average cost of treatment supplies per participant was 529 ETB (69 I$). Costs associated with delivering the intervention as part of the trial included: salaries (remuneration for community assistants and administrator costs); storage of treatment supplies; transportation of treatment supplies, community engagement activities (two sensitization workshops), community podoconiosis activities (monthly meetings with patients), and training community assistants (two training meetings). The total cost of the intervention per patient was 2,419 ETB (314 I$) with training community assistants, and 2.379 ETB (309 I$) without training. It should be mentioned that it was not possible to completely separate research costs from the intervention delivery costs, therefore the latter may be inflated.

### Dataset characteristics

Information about the completeness of the health economics dataset is summarised in S1 Appendix. Although the proportion of missing items for each variable was not large (0.3%-21.5%), the complete dataset for health economics analysis was obtained for only 323 (from 650 total) participants (immediate group n = 120, delayed group n = 203). The complete dataset comprised 50% of the total sample (immediate group n = 331, delayed group n = 329). To minimise bias introduced by missing data, multiple imputation was carried out as described in the Methods. The comparison of imputed and non-imputed datasets is shown in S2 Appendix.

### Health and social outcomes

Costs and outcomes used in the cost effectiveness analyses are summarised in Table 2. The frequency of ADLA episodes was lower in the immediate treatment group compared to the control group at 3, 6, 9 and 12 months, with a statistically significant difference at 3 and 12 months. The average number of ADLA episodes per year was 14.0 (SD 6.4) for the immediate treatment group and 12.5 (SD 4.6) for the control group.

**Table 2:** Outcomes and costs used in cost-effectiveness analysis.

Outcome	Control		Immediate treatment		P-Value (Mann-Whitney test)
	N	mean (SD)	N	mean (SD)	
**ADLA episodes/quarter**					
Baseline	317	2.79 (1.83)	303	3.01 (3.01)	0.95
3 months	309	3.47 (2.58)	267	3.02 (2.12)	0.00
6 months	295	3.20 (2.65)	240	3.04 (2.09)	0.91
9 months	294	3.27 (2.73)	223	2.99 (2.34)	0.12
12 months	285	3.21 (2.10)	225	2.61 (1.54)	0.00
DLQI*					
Baseline	329	10.22 (5.59)	321	10.92 (6.24)	0.19
3 months	329	10.52 (7.13)	321	9.03 (6.72)	0.00
6 months	329	11.26 (6.55)	321	8.92 (7.00)	0.00
9 months	329	12.04 (5.87)	321	8.72 (6.71)	0.00
12 months	329	11.38 (6.29)	321	8.80 (6.87)	0.00
WHODAS 2.0*					
Baseline	329	23.36(10.32)	321	24.69 (10.21)	0.13
12 months	329	22.04 (9.53)	321	21.76 (10.73)	0.96
**Days totally unable to work/month**					
Baseline	329	5.58 (4.30)	321	5.65 (4.11)	0.75
12 months	329	5.08 (3.90)	321	4.38 (3.93)	0.00
**Days with reduced activity/month**					
Baseline	329	4.59 (4.43)	321	4.55 (4.50)	0.45
12 months	329	3.86 (3.30)	321	3.59 (3.47)	0.06
**Cost of productivity loss/year, ETB**					
Baseline	329	321 (238)	321	323 (227)	0.99
12 months	329	286 (205)	321	252 (208)	0.00

*Lower DLQI and WHODAS 2.0 scores indicate better outcome. DLQI, Dermatology Life Quality Index; WHODAS 2.0, WHO Disability Assessment Schedule 2.0; ADLA, acute dermatolymphangioadenitis; ETB, Ethiopian Birr

DLQI scores were lower in the immediate treatment group at all time-points following the introduction of the intervention; all differences in the mean DLQI scores were statistically significant. The mean DLQI scores calculated using the area-under-the-curve method were 9.13 (SD 4.15) for the immediate treatment group and 11.16 (SD 3.71) for the control group. The differences between intervention and control groups remained apparent when sub-analyses by gender, disease stage and *kebele* location, but were more pronounced among men than women, individuals with early stages of lymphoedema (disease stage ≤2) and those who had never attended school than those who had (Table 3).

**Table 3:** Subgroup analyses using DLQI.

Variable	Control		Immediate treatment		P-Value (Mann-Whitney test)
	mean	SD	mean	SD	
**Male**					
Baseline	10.21	5.65	11.17	6.52	0.20
3 months	10.82	7.30	9.07	6.85	0.01
6 months	11.46	6.47	8.22	6.88	0.00
9 months	12.29	6.17	8.60	6.89	0.00
12 months	10.98	6.62	8.45	6.90	0.00
**Female**					
Baseline	10.24	5.50	10.60	5.90	0.57
3 months	10.18	6.90	10.00	6.57	0.15
6 months	11.04	6.62	10.00	7.03	0.08
9 months	11.75	5.47	9.00	6.49	0.00
12 months	11.84	5.85	10.00	6.79	0.00
**Disease stage ≤2**					
Baseline	10.20	5.48	10.88	6.20	0.23
3 months	10.36	7.17	8.97	6.78	0.01
6 months	11.22	6.60	8.92	7.11	0.00
9 months	12.07	5.87	8.76	6.67	0.00
12 months	11.49	6.30	9.01	6.85	0.00
**Disease stage >2**					
Baseline	10.48	6.73	12.50	6.74	0.36
3 months	12.56	6.14	10.50	5.42	0.17
6 months	11.80	5.72	9.00	4.67	0.10
9 months	11.64	5.71	8.50	7.02	0.05
12 months	10.04	5.94	2.50	6.03	0.01
**Not attended school**					
Baseline	10.42	5.52	11.08	6.27	0.26
3 months	10.71	7.26	8.54	6.51	0.00
6 months	11.28	6.48	8.61	7.00	0.00
9 months	12.15	5.79	8.56	6.45	0.00
12 months	11.33	6.35	8.84	6.83	0.00
**Attended school**					
Baseline	9.36	5.76	10.20	6.02	0.14
3 months	9.75	6.45	11.00	7.16	0.96
6 months	11.19	6.80	11.00	6.83	0.83
9 months	11.57	6.12	10.00	7.56	0.05
12 months	11.62	5.98	9.00	6.97	0.19
**Close kebele**					
Baseline	10.05	5.12	11.82	5.70	0.04
3 months	10.82	7.14	8.45	6.74	0.02
6 months	12.13	6.29	8.36	6.60	0.00
9 months	13.17	4.86	7.60	5.99	0.00
12 months	12.50	5.79	8.34	6.49	0.00
**Remote kebele**					
Baseline	10.32	5.82	10.00	6.43	0.87
3 months	10.36	7.11	10.00	6.68	0.08
6 months	10.78	6.63	9.00	7.16	0.02
9 months	11.41	6.26	10.00	6.95	0.00
12 months	10.76	6.45	9.20	7.02	0.00

Lower DLQI scores indicate better outcome. DLQI, Dermatology Life Quality Index

There were no statistically significant differences in WHODAS 2.0 scores between the immediate treatment group and the control group both at baseline and at 12 months. Subgroup analyses by gender, disease stage, *kebele* location and education also showed no statistically significant differences at 12 months (Table 4).

**Table 4:** Subgroup analyses using WHODAS 2.0.

Variable	Control		Immediate treatment		P-Value (Mann-Whitney test)
	N	mean (SD)	N	mean (SD)	
**Male**					
Baseline	22.74	11.03	24.74	10.47	0.15
12 months	22.14	10.15	21.24	10.46	0.82
**Female**					
Baseline	24.08	9.36	26.00	9.91	0.48
12 months	21.94	8.74	24.00	10.94	0.39
**Disease stage ≤2**					
Baseline	23.16	10.31	24.54	10.18	0.11
12 months	22.07	9.60	21.98	10.71	0.88
**Disease stage >2**					
Baseline	25.80	9.94	26.00	10.26	0.84
12 months	21.68	8.49	24.00	10.12	0.84
**Not attended school**					
Baseline	23.71	10.56	25.50	9.82	0.09
12 months	22.20	9.52	21.63	10.82	0.79
**Attended school**					
Baseline	21.92	8.99	22.50	11.00	0.87
12 months	21.37	9.45	25.00	10.24	0.66
**Close kebele**					
Baseline	23.50	9.79	24.64	9.94	0.37
12 months	23.87	7.65	22.59	8.66	0.79
**Remote kebele**					
Baseline	23.29	10.57	26.00	10.32	0.24
12 months	21.03	10.27	24.00	11.57	0.61

Lower WHODAS 2.0 scores indicate better outcome.; WHODAS 2.0, WHO Disability Assessment Schedule 2.0

Patients from the immediate treatment group had on average fewer days off work due to illness at 12 months compared to patients from the control group (Table 2). The estimated average number of days that patients were totally unable to work was 60 per year for the immediate treatment group and 64 per year for the control group. The estimated average number of days with reduced activity was 49 per year for the immediate intervention group and 51 per year for the control group. The average monthly cost of work productivity loss at 12 months was lower in the intervention group, 252 ETB (33 I$) compared to the control group, 286 ETB (37 I$). The estimated average annual cost of productivity loss was 3,452 ETB (449 I$) for the immediate treatment group and 3,644 ETB (474 I$) for the control group.

### Cost-effectiveness analysis

Results of the base-case cost-effectiveness analysis were derived for the imputed dataset using the generalised linear model. The total cost included in the cost-effectiveness analysis was calculated as the cost of work productivity loss due to illness for the control group, and the cost of intervention plus the cost of productivity loss for the immediate treatment group. The intervention cost included the cost of training community podoconiosis assistants. Cost-effectiveness analyses were carried out using the three effectiveness outcomes: the number of ADLA episodes, DLQI and WHODAS 2.0 scores. The results of the base-case cost-effectiveness analysis are summarised in Table 5.

**Table 5:** Summary of the cost-effectiveness analyses using different effectiveness outcomes.

	Total cost, ETB mean (SD)	Total effect mean (SD)	Difference in cost, ETB mean (95% CI)	Difference in effect mean (95% CI)	ICER
**ADLA episodes**					
Control	3,662 (442)	3.39 (0.26)	-22 (-86; 43)	0.18 (0.14; 0.22)	Intervention dominates
Immediate treatment	3,640 (424)	3.21 (0.26)			
DLQI*					
Control	3,662 (442)	11.17 (0.27)	-22 (-86; 43)	-2.06 (-2.10; -2.02)	Intervention dominates
Immediate treatment	3,640 (424)	9.11 (0.26)			
WHODAS 2.0*					
Control	3,662 (442)	22.6 (1.09)	-22 (-86; 43)	0.57 (0.40; 0.74)	Intervention less costly and less effective
Immediate treatment	3,640 (424)	23.2 (1.09)			

*Lower DLQI and WHODAS 2.0 scores indicate better outcome. DLQI, Dermatology Life Quality Index; WHODAS 2.0, WHO Disability Assessment Schedule 2.0; ADLA, acute dermatolymphangioadenitis; ETB, Ethiopian Birr; ICER, incremental cost-effectiveness ratio.

**Fig 1:**
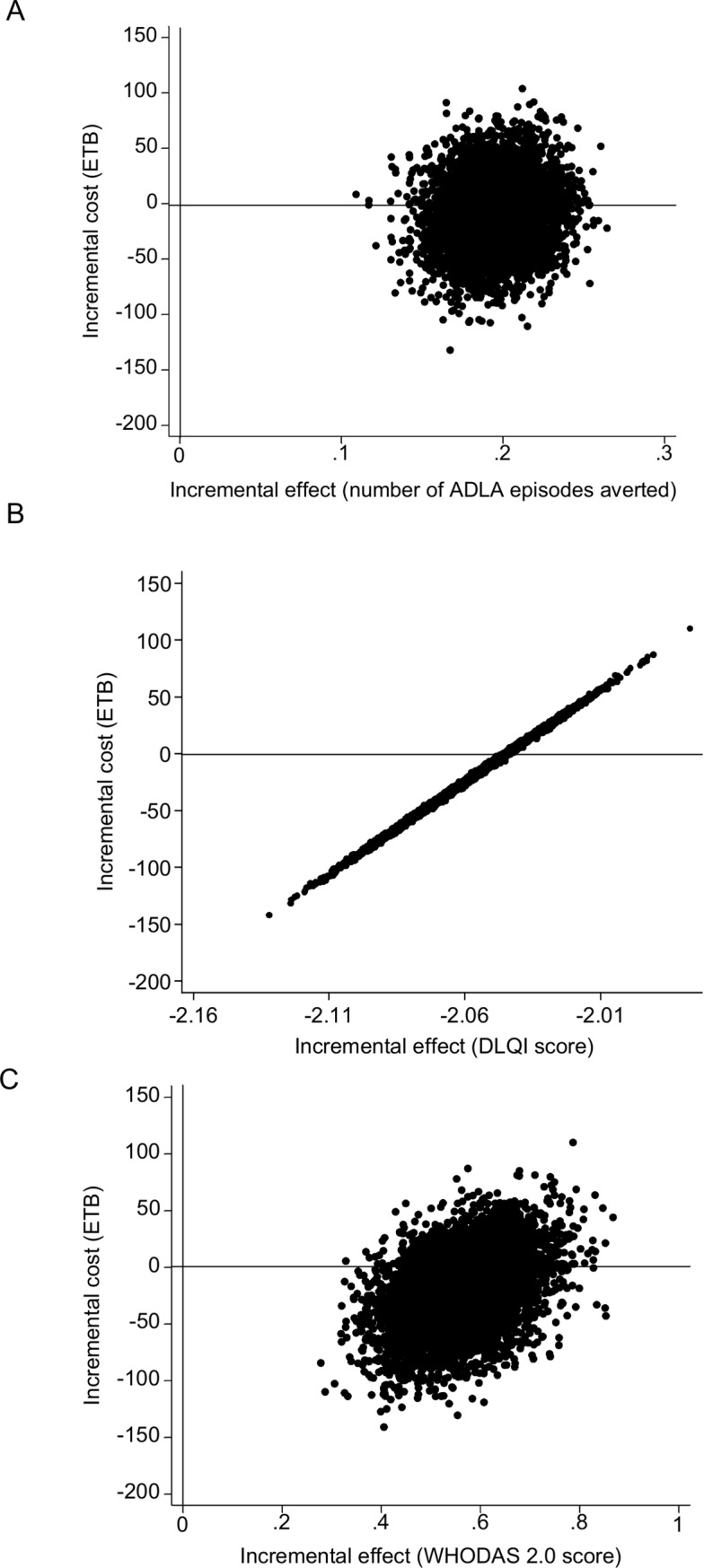
Cost-effectiveness planes generated using different effectiveness outcomes: the number of ADLA episodes averted (A), DLQI scores (B) and WHODAS 2.0 scores (C) analysed using GLM. The graph shows 5,000 bootstrap estimates. ETB, Ethiopian Birr.

Analysis based on the frequency of ADLA episodes showed that treatment was on average less costly (negative difference in cost) and more effective (negative difference in the number of ADLA episodes) compared to control. The mean difference in cost was small, 22 ETB (3 I$) and the confidence intervals were wide, indicating the possibility of the intervention being overall more costly than control. Uncertainty around the incremental cost-effectiveness ratio was assessed using the non-parametric bootstrap method. A cost-effectiveness plane depicting 5,000 bootstrap estimates is shown on Fig 1A. The graph demonstrates that in 75% of estimations the immediate treatment was less costly and more effective in reducing the frequency of ADLA episodes compared to control, and in 25% the immediate treatment was more costly and more effective compared to control.

Cost-effectiveness analysis conducted using DLQI showed similar results with immediate treatment being on average less costly and more effective than the control (Table 5). There was a statistically significant difference in the DLQI scores between the two groups. Probabilistic analysis showed that in 75% of simulations the immediate treatment was less costly and more effective compared to control Fig 1B. In the remaining 25% of simulations the immediate treatment was more costly and more effective compared to control.

Cost effectiveness analyses conducted using WHODAS 2.0 demonstrated that the intervention was less effective in reducing disability in the immediate treatment group compared to the control group, but the difference was very small (2.6% change in average scores) (Table 5). The cost-effectiveness plane shows that in 75% of simulations the immediate treatment was less effective and less costly, and in 25% of simulations the immediate treatment was less effective and more costly compared to control (Fig 1C).

Sensitivity analyses were conducted using two different models (SUR and MLM) as well as non-adjusted data (S3 Appendix). The cost-effectiveness estimates derived using different models were close, indicating the robustness of the results.

Scenario analyses were conducted to account for training community podoconiosis assistants as a part of the intervention cost (S4 Appendix). Removing training costs did not have a pronounced effect on the results of the cost-effectiveness analysis.

Subgroup analyses were carried out for patients with a range of family incomes. We separated two subgroups of patients based on their baseline family income (<1,000 ETB/month and ≥1,000 ETB/month). Results of the subgroup analyses adjusted for covariates using GLM are summarised in Table 6.

**Table 6:** Summary of the cost-effectiveness sub-group analyses based on monthly family income: <1.000 ETB and ≥1,000 ETB.

	Total cost, ETB mean (SD)	Total effect mean (SD)	Difference in cost, ETB mean (95% CI)	Difference in effect mean (95% CI)	ICER
**ADLA episodes, income <1,000 ETB**					
Control	3,594 (467)	3.41 (0.27)	-111 (-187; -35)	0.18 (0.13; 0.22)	Intervention dominates
Immediate treatment	3,483 (449)	3.23 (0.26)			
**ADLA episodes, income ≥1,000 ETB**					
Control	4,032 (615)	3.32 (0.25)	487 (241; 733)	0.21 (0.11; 0.31)	Intervention more effective and more costly
Immediate treatment	4,518 (612)	3.11 (0.25)			
DLQI*, income <1,000 ETB					
Control	3,594 (467)	11.00 (0.32)	-111 (-187; -35)	-1.9 (-1.95; -1.85)	Intervention dominates
Immediate treatment	3,483 (449)	9.09 (0.31)			
DLQI*, income ≥1,000 ETB					
Control	4,032 (615)	12.14 (0.63)	487 (241; 733)	-3.19 (-3.47; -2.92)	Intervention more effective and more costly
Immediate treatment	4,518 (612)	8.95 (0.73)			
WHODAS 2.0*, income <1,000 ETB					
Control	3,594 (467)	22.2 (1.44)	-111 (-187; -35)	0.90 (0.67; 1.14)	Intervention less effective and less costly
Immediate treatment	3,483 (449)	23.11 (1.38)			
WHODAS 2.0* **, income ≥1,000 ETB**					
Control	4,032 (615)	24.71 (1.06)	487 (241; 733)	-0.93 (-1.38; -0.49)	Intervention more effective and more costly
Immediate treatment	4,518 (612)	23.77 (1.11)			

*Lower DLQI and WHODAS 2.0 scores indicate better outcome. DLQI, Dermatology Life Quality Index; WHODAS 2.0, WHO Disability Assessment Schedule 2.0; ADLA, acute dermatolymphangioadenitis; ETB, Ethiopian Birr; ICER, incremental cost-effectiveness ratio.

**Fig 2:**
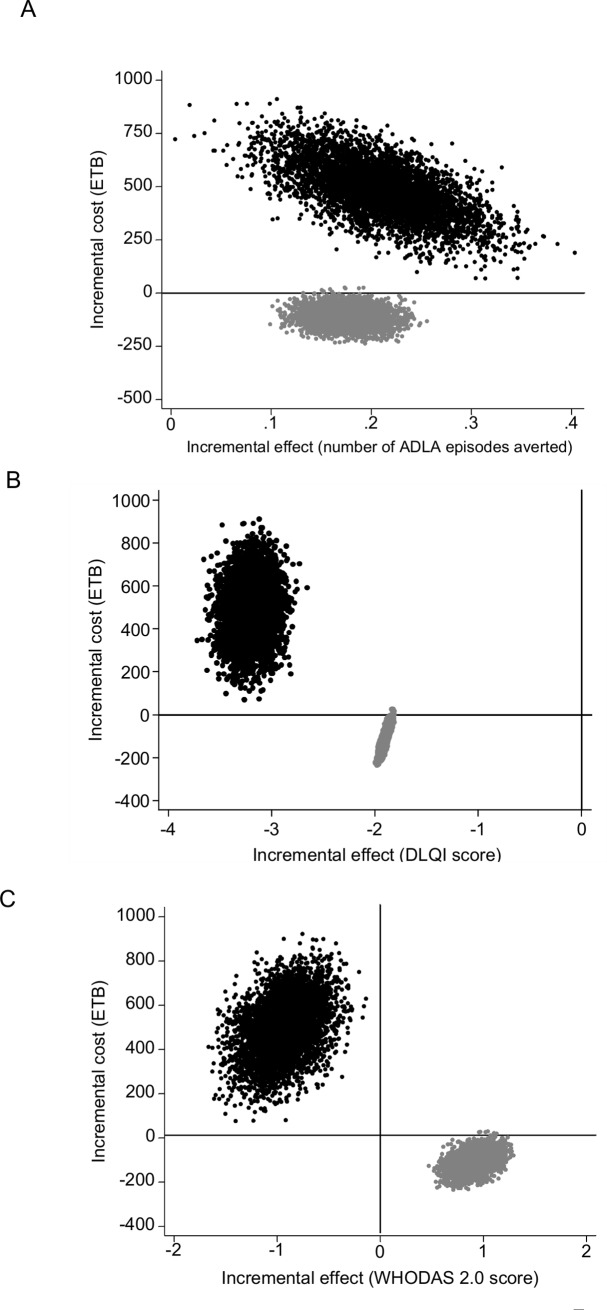
Cost-effectiveness planes for patients with monthly family income <1,000 ETB (grey circles) and monthly family income ≥1,000 ETB (black circles). The graphs show 5,000 bootstrap estimates for the number of ADLA episodes (A), DLQI (B) and WHODAS 2.0 (C). ETB, Ethiopian Birr.

For patients with lower family income the immediate treatment was less costly and more effective than control in reducing the number of ADLA episodes. For patients with higher family income, the immediate treatment was more costly and more effective than control, but the difference in effectiveness was only marginally higher from that for the low income group. The cost-effectiveness plane illustrates that differences between the two subgroups were driven by the cost of productivity loss (Fig 2A). Differences in the cost-effectiveness between the two income subgroups were even more pronounced when analyses were conducted using DLQI and WHODAS 2.0 (Table 6, Fig 2B and 2C). For the subgroup with low family income the immediate treatment was cost-effective (both less costly and more effective compared to the control) in reducing the number of ADLA episodes and improving health-related quality of life, but less effective compared to control in reducing disability. For the subgroup with higher family income the intervention was more effective but more costly than control for all effectiveness outcomes.

## Discussion

This is the first study to assess the cost and cost-effectiveness of a lymphoedema management intervention for people affected by podoconiosis. In the context of a pragmatic randomised controlled trial, the package of care was effective in reducing the incidence of ADLA episodes with statistically significant differences at 3 and 12 months after introducing the intervention. Disease-specific quality of life measured using DLQI was higher in the intervention group at all data points. We did not find any significant change in disability (WHODAS 2.0) by the end of the intervention, overall, or by subgroup. The previous, uncontrolled follow-up study [8] also demonstrated marked (positive) changes in DLQI score (WHODAS was not used in that study). These changes occurred rapidly, before significant physical changes were observed, suggesting that quality of life changes are driven by social and emotional factors more than physical changes. WHODAS 2.0 is less specific about pain, embarrassment and problems with partners, close relatives and friends than DLQI, and it is possible that this is why we did not see significant change in WHODAS 2.0 scores.

Our recent process evaluation study (S5 Appendix) explored patients' perceptions of the intervention and the impact it had through reducing the frequency of ADLA. Seventy-three patients who had completed the trial, 10 family members and 8 Community Podoconiosis Agents took part in Focus Group Discussions or In-Depth Interviews. Overwhelmingly, process evaluation participants considered the intervention to be effective in terms of improving the health, economic and social aspects of patients’ lives, particularly through impact on the frequency, duration and severity of acute attack episodes. Two representative quotes are given below. The interviewer's impression was that even small numerical reductions in ADLA frequency made important differences to patients' lives. Quotes:

“*Previously*, *I used to have mitchader [acute attack] day-after-day…but now*, *I have it may be once a month only…before*, *we used to lag behind on tasks such as crop gathering*, *but now we finish at the same time as others…why*, *because we’re healthy” (FGD*, *P010*, *patient*, *male)*.

*“Before she started the treatment*, *her feet used to get puffed-up*, *now that’s no more…previously; she used to get ill [acute attack] everyday …she recently got ill*, *but she got better immediately and the swelling*, *has decreased very much… wearing shoes helped reduce the swelling” (IDI*, *P061*, *family member*, *male)”* (S5 Appendix).

The cost of community-based lymphoedema management was mainly driven by costs associated with delivery of the intervention (transportation, staff salaries, community activities and training). The cost of hygiene and treatment supplies comprised 22% of the total cost of the intervention. Given that lymphoedema management intervention was a part of a randomised controlled trial, there were up-front costs associated with setting up the delivery of care. We expect the cost of the intervention to go down if it is absorbed into standard government provision.

Results of cost-effectiveness analyses demonstrate that in three-quarters of estimations the community-based intervention was more effective and less costly than not intervening, due to decrease in frequency of ADLA episodes, improved health-related quality of life and reduced work productivity losses. Our analyses suggest that some subgroups of patients would benefit more from the intervention, including people who had not attended the school and those living on lower incomes.

The only other study to estimate costs and benefits of lymphoedema management examined a large community-based programme (‘CASA’) in Odisha, India [29]. A non-government organisation used community health workers to train patients with LF lymphoedema in leg washing and use of topical antibiotic and antifungal treatments. While the intervention was similar to that trialled in GoLBeT, it was not identical, for example, bandaging was not used in India, and socks and shoes were not provided. The programme evaluation, based on an economic model, showed per-person savings to be more than 130 times the per-person costs of the programme. There are several explanations for the much greater cost-benefits of this LF lymphoedema programme, including the programme being long-established (>10 years) and the impact on ADLA was extrapolated from observational studies [30]. The model also included cost of medical care and out-of-pocket expenses for people with LF [29]. A life-time horizon was assumed in the model, and assumptions were made concerning LF morbidity, life expectancy and the labour market. The cost of the programme per patient was I$10.00- I$12.50 over 24 months. It is unclear how these costs were calculated and which costing categories were included [31].

Unlike the CASA economic study, the results of our analyses were based on individual-level data, allowing for possible correlations between costs and health outcomes for each participant. Out-of-pocket medical costs were not considered. Cost of intervention per patient was calculated over one year and included programme set-up costs. Uncertainty in costs and outcomes was assessed using non-parametric methods reflecting real-life probability distributions.

An earlier, uncontrolled study among podoconiosis patients demonstrated highly significant improvement in quality of life with treatment [8], as did a preliminary study among patients with LF lymphoedema in Guyana [32]. In each case, quality of life changes outstripped clinical changes, suggesting that other, possibly social factors were as important as clinical effects in improving reported quality of life.

We observed no significant difference between intervention and control groups in reduction in disability within the 12-month follow-up of GoLBeT. Uncontrolled follow-up of LF patients on the CASA treatment programme over 24 months demonstrated significant reductions in disability, also measured using WHODAS 2.0 [33]. The differences may be explained by the different study designs. In the CASA programme, the greatest improvements in total disability score were seen at 6 months, with slight rebound at 12 and 24 months, so duration of follow-up does not seem to explain the difference between studies.

### Limitations

The analyses of social and economic outcomes presented in this study have a number of limitations:

1. Economic evaluation was not planned as a part of this study, therefore costs to other individuals (e.g. care givers) and health care system (government clinics) were not included in the economic analyses. There was no government provision of podoconiosis lymphoedema care at the time. However, our results show the community-based lymphoedema management to be cost effective even when work productivity costs alone are taken into account.
2. It was not always possible to separate costs associated with intervention and research (e.g. transportation and workshops). Therefore, the cost of intervention reported in this study may be inflated.
3. The follow-up period was quite short (12 months), therefore we were unable to capture long-term effects of the intervention on work productivity and disability. Longer term follow-up seems necessary to capture changes in reported disability and is advised for future studies.

### Conclusions

The results of our economic evaluation suggest that the community-based lymphoedema intervention can be cost-effective in reducing the frequency of ADLA episodes, improving health-related quality of life and decreasing work productivity losses. In three-quarters of estimations, the intervention appeared to be cost-saving due to improved work productivity. Subgroups of patients who had not attended the school and those living on low income seem to benefit the most from this intervention.

## Supporting information

S1 Appendix Completeness of health economics data (%). (DOCX)

S2 Appendix Comparison of non-imputed and imputed datasets. (DOCX)

S3 Appendix Summary of the cost-effectiveness sensitivity analyses using different models. (DOCX)

S4 Appendix Summary of the cost-effectiveness scenario analyses. (DOCX)

S5 Appendix Process evaluation of a randomized controlled trial to test the effectiveness of a simple foot care and hygiene intervention in podoconiosis lymphoedema in northern Ethiopia. (DOCX)
