# Supplementary material for: Cost-effectiveness and social outcomes of a community-based treatment for podoconiosis lymphoedema in the East Gojjam zone, Ethiopia
Source: PLoS Negl Trop Dis. 2019 Oct 23;13(10):e0007780. doi: 10.1371/journal.pntd.0007780 (PMC6808421; doi:10.1371/journal.pntd.0007780)
Supplement: S1 Appendix — (DOCX) [file pntd.0007780.s001.docx]

**Appendix 1.** Completeness of health economics data (%)

| **Variable** | **Baseline** | **3 months** | **6 months** | **9 months** | **12 months** |
| --- | --- | --- | --- | --- | --- |
| **DLQI** | 95.9 | 99.4 | 99.2 | 99.4 | 99.2 |
| **WHODAS 2.0** | 97.7 | N/A | N/A | N/A | 100 |
| **ADLA episodes** | 95.4 | 88.6 | 82.3 | 79.5 | 78.5 |
| **Days totally unable to work** | 98.9 | N/A | N/A | N/A | 93.8 |
| **Days with reduced activity** | 100 | N/A | N/A | N/A | 85.5 |
| **Income** | 99.1 | N/A | 99.7 | N/A | 100 |

N/A, not applicable (data were not collected)
