# Supplementary material for: Cost-effectiveness and social outcomes of a community-based treatment for podoconiosis lymphoedema in the East Gojjam zone, Ethiopia
Source: PLoS Negl Trop Dis. 2019 Oct 23;13(10):e0007780. doi: 10.1371/journal.pntd.0007780 (PMC6808421; doi:10.1371/journal.pntd.0007780)
Supplement: S2 Appendix — (DOCX) [file pntd.0007780.s002.docx]

**Appendix 2**. Comparison of non-imputed and imputed datasets

| **Data** | **Control**  **mean (SD)** | | | **Immediate treatment**  **mean (SD)** | |
| --- | --- | --- | --- | --- | --- |
|  | **Non-imputed** | **Imputed** | | **Non-imputed** | **Imputed** |
| **DLQI** | | | | | |
| Baseline | 10.26 (5.58) | 10.22 (5.59) | | 10.94 (6.23) | 10.91 (6.24) |
| 3 months | 10.55 (7.12) | 10.52 (7.13) | | 9.06 (6.71) | 9.03 (6.72) |
| 6 months | 11.27 (6.53) | 11.26 (6.55) | | 8.95 (7.01) | 8.92 (7.00) |
| 9 months | 12.05 (5.87) | 12.04 (5.87) | | 8.75 (6.27) | 8.72 (6.71) |
| 12 months | 11.37 (6.31) | 11.38 (6.29) | | 8.86 (6.78) | 8.80 (6.87) |
| **WHODAS 2.0** | | | | | |
| Baseline | 23.23 (10.33) | | 23.37 (10.32) | 24.69 (10.17) | 24.69 (10.21) |
| 12 months | 22.04 (9.53) | | N/A | 21.76 (10.73) | N/A |
| **Days totally unable to work** | | | | | |
| Baseline | 5.59 (4.32) | 5.58 (4.30) | | 5.66 (4.12) | 5.65 (4.11) |
| 12 months | 5.16 (3.96) | 5.08 (3.90) | | 4.49 (4.06) | 4.38 (3.93) |
| **Days with reduced activity** | | | | | |
| Baseline | 4.59 (4.43) | N/A | | 4.55 (4.50) | N/A |
| 12 months | 3.82 (3.38) | 3.86 (3.30) | | 3.74 (3.71) | 3.59 (3.47) |

N/A, not applicable (100% complete). The numbers of ADLA episodes were not imputed
