# Supplementary material for: Cost-effectiveness and social outcomes of a community-based treatment for podoconiosis lymphoedema in the East Gojjam zone, Ethiopia
Source: PLoS Negl Trop Dis. 2019 Oct 23;13(10):e0007780. doi: 10.1371/journal.pntd.0007780 (PMC6808421; doi:10.1371/journal.pntd.0007780)
Supplement: S3 Appendix — (DOCX) [file pntd.0007780.s003.docx]

**Appendix 3.** Summary of the cost-effectiveness sensitivity analyses using different models

|  | **Difference in cost, ETB**  **mean (95% CI)** | **Difference in effect**  **mean (95% CI)** | **ICER** |
| --- | --- | --- | --- |
| **ADLA episodes averted** | | | |
| Not adjusted | 11 (-288; 310) | 0.24 (-0.32; 0.51) | Intervention more effective and more costly |
| GLM | -22 (-86; 43) | 0.18 (0.14; 0.22) | Intervention dominates |
| SUR | 11 (49; 72) | 0.19 (0.15; 0.23) | Intervention more effective and more costly |
| MLM | -10 (-245; 233) | 0.24 (0.18; 0.30) | Intervention dominates |
| **DLQI*** | | | |
| Not adjusted | 11 (-288; 310) | -2.03 (-2.63; -1.42) | Intervention more effective and more costly |
| GLM | -22 (-86; 43) | -2.06 (-2.10; -2.02) | Intervention dominates |
| SUR | 11 (49; 72) | -2.03 (-2.06; -1.99) | Intervention more effective and more costly |
| MLM | -10 (-245; 233) | -2.01 (-2.18; -1.85) | Intervention dominates |
| **WHODAS 2.0*** | | | |
| Not adjusted | 11 (-288; 310) | 0.52 (-0.52; 1.56) | Intervention less effective and more costly |
| GLM | -22 (-86; 43) | 0.57 (0.40; 0.74) | Intervention less effective and less costly |
| SUR | 11 (49; 72) | 0.52 (0.35; 0.69) | Intervention less effective and more costly |
| MLM | -10 (-245; 233) | 0.54 (-0.18; 1.24) | Intervention less effective and less costly |

*Lower DLQI and WHODAS 2.0 scores indicate better outcome. ETB, Ethiopian Birr
