# Supplementary material for: Cost-effectiveness and social outcomes of a community-based treatment for podoconiosis lymphoedema in the East Gojjam zone, Ethiopia
Source: PLoS Negl Trop Dis. 2019 Oct 23;13(10):e0007780. doi: 10.1371/journal.pntd.0007780 (PMC6808421; doi:10.1371/journal.pntd.0007780)
Supplement: S4 Appendix — (DOCX) [file pntd.0007780.s004.docx]

**Appendix 4.** Summary of the cost-effectiveness scenario analyses

|  | **Difference in cost, ETB**  **mean (95% CI)** | **Difference in effect**  **mean (95% CI)** | **ICER** |
| --- | --- | --- | --- |
| **ADLA episodes averted** | | | |
| Including training | -22 (-86; 43) | 0.18 (0.14; 0.22) | Intervention dominates |
| Excluding training | -25 (-89; 40) | 0.18 (0.14; 0.22) | Intervention dominates |
| **DLQI*** | | | |
| Including training | -22 (-86; 43) | -2.06 (-2.10; -2.02) | Intervention dominates |
| Excluding training | -25 (-89; 40) | -2.06 (-2.10; -2.02) | Intervention dominates |
| **WHODAS 2.0*** | | | |
| Including training | -22 (-86; 43) | 0.57 (0.40; 0.74) | Intervention less effective and less costly |
| Excluding training | -25 (-89; 40) | 0.57 (0.40; 0.74) | Intervention less effective and less costly |

*Higher DLQI and WHODAS 2.0 scores indicate greater disability. Data were adjusted for covariates using GLM. ETB, Ethiopian Birr
