# Supplementary material for: Cost-effectiveness and social outcomes of a community-based treatment for podoconiosis lymphoedema in the East Gojjam zone, Ethiopia
Source: PLoS Negl Trop Dis. 2019 Oct 23;13(10):e0007780. doi: 10.1371/journal.pntd.0007780 (PMC6808421; doi:10.1371/journal.pntd.0007780)
Supplement: S5 Appendix — (DOCX) [file pntd.0007780.s005.docx]

**Appendix 5.**

**Process evaluation of a randomized controlled trial to test the effectiveness of a simple foot care and hygiene intervention in podoconiosis lymphoedema in northern Ethiopia**

Henok Negussie^1†^, Ayenew Negesse^2^, Meseret Molla Kassahun^3^, Gail Davey^1*^

^1^Wellcome Trust Centre for Global Health Research, Brighton and Sussex Medical School, University of Sussex, Falmer, Brighton, BN1 9PX

^2^College of Medicine and Health Sciences, Debre Markos University, Debre Markos, Ethiopia

^3^Department of RH and Health Services, School of Public Health, College of Health Sciences, Addis Ababa University, Addis Ababa, Ethiopia

† Died suddenly in February 2018

* Corresponding author

Tel: +44-1273-872528

Emails: [ayenewnegesse@gmail.com](mailto:ayenewnegesse@gmail.com); [mesenana_03@yahoo.com](mailto:mesenana_03@yahoo.com); [g.davey@bsms.ac.uk](mailto:g.davey@bsms.ac.uk)

**Abstract**

Background: Process evaluations can offer valuable insights when interpreting the results of randomised controlled trials. This study was conducted alongside a trial of a foot care and hygiene intervention for podoconiosis lymphoedema (leg swelling attributable to a mineral rather than parasitic cause). It aimed to examine whether the intervention was delivered as planned, and to explore participants’ and providers’ experiences of and responses to the intervention.

Methods: This was a process evaluation nested within a pragmatic randomised controlled trial of podoconiosis treatment (a complex intervention comprising use of water and soap, Whitfield ointment, bandages, exercises, socks and shoes). A total of 98 participants (patients enrolled in the trial, family members, Community Podoconiosis Assistants, intervention supervisors, Health Extension Workers and a *Woreda* (District) Health Office expert) were included in Focus Group Discussions and In-depth Interviews. Semi-structured interview guides were developed to examine perceived effectiveness, fidelity, feasibility, satisfaction and sustainability of the foot care and hygiene intervention. Direct content analysis was conducted. This trial within this process evaluation was nested was registered with the International Standard Randomised Controlled Trials Number Register, number ISRCTN67805210 on 24/01/2013.

Results: Participants reported that the intervention reduced the frequency of acute attacks and improved social and economic aspects of patients’ lives. Non-adherence was uncommon, but was related to shortage of water, travelling for religious purposes, and quality of intervention products. Participants recommended that future treatment be provided under conditions similar to the trial, as health care staff are already overburdened and Health Centers are often far from patients’ homes. Participants recommended addition of antibiotics and analgesics to treat acute attacks in future programs.

Conclusion: The foot care and hygiene intervention was acceptable and was reported to bring about health, social and economic improvements. It was understood and correctly practiced by most patients and considered feasible by most participants, though several questions around sustainability were raised. Implementation in other contexts should take into account availability of water and treatment supplies.

**Key words: Pragmatic Randomised Clinical trials, lymphoedema management, podoconiosis, process evaluation, adherence**

**Background**

Podoconiosis is a form of lymphoedema (leg swelling) arising in people going barefoot in highland tropical areas[[1](#_ENREF_1)]. It is a significant problem in tropical Africa where irritant soils have been generated by environmental conditions of high altitude (>1,000m) and high rainfall (>1,000mm per annum) and are farmed by very poor communities. The disease leads to significant stigma, reduced productivity and quality of life[[2-5](#_ENREF_2)]. However, misdiagnosis (chiefly confusion with filarial lymphoedema) and fatalism have hampered treatment [[6](#_ENREF_6)].Only in the past two decades has foot hygiene treatment[[7](#_ENREF_7)] been offered specifically to people with podoconiosis[[8](#_ENREF_8)], and this has been initiated by non-government organisations without state adoption or systematic evaluation. Based on the urgent need for evidence on which to base national policy, a fully randomised controlled trial of the intervention was conducted in northern Ethiopia[[9](#_ENREF_9)].

Randomised controlled trials (RCTs) are the most rigorous way to evaluate the effectiveness of interventions, regardless of their complexity. Pragmatic RCTs provide high quality ways of investigating the effectiveness of treatments, interventions, or other aspects of healthcare provision under ‘real life’ (rather than highly controlled) conditions[[10](#_ENREF_10)]. Process evaluations within RCTs ask important questions relevant to their execution and context which often facilitate interpretation of results. They may aim to examine the views of participants on the intervention, to study how the intervention is implemented, to distinguish between components of the intervention, investigate contextual factors that may affect an intervention or study the way effects may vary in subgroups[[11](#_ENREF_11), [12](#_ENREF_12)]. While RCTs are intended to examine the effectiveness of an intervention, more nuanced understanding of ‘what works?’, ‘for whom?’ and ‘under what circumstances?’ is becoming increasingly important for health service interventions to be useful in informing wider implementation[[13](#_ENREF_13)]. Process evaluations can provide in-depth understanding of the acceptability of interventions and whether they will work in everyday life. This information is vital for potential service users and providers, and to policy makers concerned with whether interventions can and should be implemented on a larger scale[[14](#_ENREF_14)]. While generally considered to be useful in understanding trial implementation and outcomes, process evaluations are susceptible to bias. The main biases likely are selection bias (respondents with positive views on the trial being consciously or unconsciously selected) and social desirability bias (respondents offer views they think will be considered favourably by others, for example, the interviewer)[[15](#_ENREF_15)].

The Gojjam Lymphoedema Best Practice Trial (GoLBeT) was a pragmatic Randomised Controlled Trial with two arms designed to evaluate the effectiveness of podoconiosis lymphoedema management in the community. This trial was registered on 24/01/2013 with the International Standard Randomised Controlled Trials Number Register, number ISRCTN67805210. The RCT set out to test the hypothesis that community-based treatment of podoconiosis lymphoedema reduces the frequency of acute dermatolymphangioadenitis episodes (ADLA, ‘acute attacks’) and secondarily that it improves other clinical, social and economic outcomes. Podoconiosis patients aged 18 years and older were individually randomised to a package comprising instruction in foot hygiene, skin care, bandaging, exercises, use of socks and shoes, with support by lay Community Podoconiosis Agents (CPAs) at Monthly Intervention Meetings; or no intervention. Full details of the RCT protocol and the Rapid Ethical Assessment that preceded recruitment are described in previous articles[[9](#_ENREF_9), [16](#_ENREF_16)]. The primary outcome results have been published [[17](#_ENREF_17)], as has detailed description of the screening and enrolment process[[18](#_ENREF_18)]. Here we present the results of a process evaluation whose objectives were to examine whether the intervention was delivered as intended, and to explore staff and participants’ responses to and experiences of the intervention. We anticipate that this process evaluation will identify issues that may need to be considered in scaling up the intervention in other contexts.

**Methods**

Study setting: The study was nested in (ran parallel to, during the second half of) a RCT of podoconiosis treatment in northern Ethiopia (GoLBeT). The RCT was conducted in Aneded *woreda* (district), East Gojjam Zone, Amhara Regional State where prevalence of podoconiosis is 3.4% in the adult population[[19](#_ENREF_19)]. The *woreda* is located 300 Kms North West of Addis Ababa and 20 Kms South East of Debre Markos, the zonal capital. According to the 2007 Census, Aneded *woreda* is made up of 19 *kebeles* (smallest administrative unit) and has a total population of 91,224, with 89,446 living in rural *kebeles*. It has four Health Centres. During selection of participants for the RCT, patients already undertaking self-treatment comparable to the intervention were excluded. The intervention phase of the RCT ran from February 2015 until August 2016. Full details of participant selection and the trial intervention are available in the published RCT protocol[[9](#_ENREF_9)], a flow chart for screening and recruitment in the article summarizing these [[18](#_ENREF_18)] and the RCT flow chart in the main outcome article[[17](#_ENREF_17)].

**Figure 1 Map of Ethiopia showing East and West Gojjam zones, Amhara Regional State. Credit: Oxford University Press**

Study design: A process evaluation was carried out from February to June 2016. This was based on a logic model developed during preparation for the RCCT, which defined the key components of the intervention and how they were expected to interact with each other and the surrounding context. We developed the process evaluation to focus on explaining any intervention effects, and to explore how the intervention was implemented and received by patients, their family members and relevant health professionals. Study population: In-Depth Interviews and Focus Group Discussions were conducted among patients enrolled in the RCT and their families, Community Podoconiosis Assistants (CPAs – women employed by the RCT to train patients to manage their legs), their supervisors, and Health Extension Workers (HEWs) in selected study *kebeles*. Focus Group Discussions were held when patients were already grouped at Monthly Intervention Meetings in their sub-districts, or CPAs were already gathered for a supervision meeting. In-depth interviews were held when specific perspectives were required, and groups representing these perspectives were not already meeting. Invitations to patients to take part in Focus Group Discussions were made orally by CPAs at the beginning of a Monthly Intervention Meeting in a given *kebele*. The Focus Group Discussions were held once the patients had washed their feet and had their supplies replenished. Any patient that agreed to stay after the meeting was included in the FGD. At the end of the discussions, invitations for family members to participate in in-depth interviews were issued through patients. CPAs were asked to take part in a FGD after one of their regular supervision sessions, and all that agreed were included. Patients who had completed their 12 months in the RCT were approached in a *kebele* in which no patient FGD had been conducted. The sole patient who had voluntarily withdrawn was approached by their local CPA in another sub-district not represented in the Focus Group Discussions. The role of the Trial Coordinator was to ensure a wide range of sub-districts was represented (14 of 19 were represented). Refusal to participate and withdrawal from the study once discussion had commenced were not encountered. The selection of participants was an iterative process: once the preliminary interviews had been reviewed and analyzed, further data collection was arranged and new participants identified. Data collection continued to the point of saturation.

Study tools: semi-structured interview guides were developed based on the framework recommended by the UK Medical Research Council[[20](#_ENREF_20), [21](#_ENREF_21)]. Interviews with patients explored the following: acceptability and feasibility of the intervention; adherence to and unanticipated consequences of the intervention; perceived impacts of the intervention (health, economic and social); satisfaction with the way the intervention was delivered; barriers to implementing the intervention at home, and improvements required for future treatment and sustainability. Discussion guides were translated into Amharic (the local language). Interviews with CPAs explored: adherence with the intervention; barriers to practicing the intervention and likely implications for translation into routine policy and practice. In addition one Key Informant Interview was conducted with a *Woreda* Health Office expert.

Data collection: Interviews were carried out in Amharic. With the permission of participants, all Focus Group Discussions and In-Depth Interviews were audio-recorded. This was supplemented by simultaneous note-taking during discussions. Interviews and Focus Group Discussions were led by a male public health expert with considerable qualitative research and cultural experience in the region but no involvement in the RCT (AN). The Trial Coordinator (HN) was involved in the development of interview schedules and in clarifying issues that arose in the field during debriefing sessions held after each field activity. Participants were clearly informed of the purpose of the focus groups and interviews and rules of discussion clarified. The moderator was introduced to participants as an independent assessor of the intervention, to encourage them to discuss their true perceptions and practices freely. To control dominance during discussions, speaking orders were varied to encourage shy participants and control stronger voices. Analysis of the first few Focus Group Discussions gave no indication of a particular order of responses to the questions. Venues conducive to discussion were used to ensure privacy.

Data analysis: A log sheet was used to document data collection activities. All data collected, (including interview tapes, transcribed and translated material, and field notes) were constantly checked, indexed and systematically categorized. Data analysis was initiated alongside data collection. First, all interviews and Focus Group Discussions were transcribed verbatim, translated into English and checked for consistency. This was followed by close reading of the transcripts and coding. HN conducted coding with discussion and agreement with AN on interpretation and meaning. The coding was based on the research question concerning key components of the intervention. Data analysis was performed using a directed content analysis[[22](#_ENREF_22)] based on the MRC process evaluation guidance[[23](#_ENREF_23)] and prior research on relevant issues from the podoconiosis literature (including barriers to access to care[[24](#_ENREF_24)], discontinuation of treatment[[25](#_ENREF_25), [26](#_ENREF_26)], and barriers to shoe use[[27](#_ENREF_27)]) and the lymphatic filariasis literature[[28-31](#_ENREF_28)]. The following five key themes were the focus of analyses: perceived impacts of the intervention; fidelity, feasibility; satisfaction with the way the intervention was delivered; and sustainability. Data were organized using NVivo 11 Pro for Windows.

Ethical considerations: Ethical approval for the study was obtained from the Ethics Review Committee of the Amhara Regional Health Bureau (reference number HRTT/11/127/08; approval date: 22/01/2016). While participants in the RCT had given written informed consent[[17](#_ENREF_17)], participants in the process evaluation gave oral consent to participate, which was recorded. Participants were also informed that any information they provided would be kept confidentially and that the final report would not include personally-identifiable information. Interview tapes will be destroyed after five years of completion of the study (as per University of Sussex policies).

**Results**

A total of 98 participants, 53 females and 45 males took part in the process evaluation. These included 73 patients from the intervention group, ten non-affected family members, eight CPAs, two CPA supervisors, three HEWs, and a Woreda Health Office expert. The majority were over the age of 40 years, married and farmers (Table 1).

**Table 1: Participants’ socio-demographic characteristics**

| **Characteristic** | **Number of participants** |
| --- | --- |
| Sex | |
| Male | 45 |
| Female | 53 |
| Age | |
| 18-29 | 22 |
| 30-39 | 7 |
| 40-49 | 26 |
| 50-59 | 28 |
| 60+ | 15 |
| Marital Status | |
| Married | 76 |
| Widowed | 7 |
| Divorced | 1 |
| Single | 14 |
| Occupation | |
| Farmer (patients) | 73 |
| Farmer (non-affected family members) | 10 |
| Community Podoconiosis Agent | 8 |
| Supervisor | 2 |
| HEW | 3 |
| WoHO Officer | 1 |
| Liquor seller | 1 |

Ten Focus Group Discussions were held with patients who were still part of the RCT and CPAs, while 19 In-Depth Interviews were conducted with family members, HEWs, CPA supervisors, a Woreda Health Office expert, patients who had completed treatment and patients who had voluntarily left the study. Participants from 14 of the 19 *kebeles* in which the RCT was conducted were included (Table 2).

**Table 2: Data collection activities and number of participants by study *kebele***

| **In-depth Interviews** | | | **Focus Group Discussions** | | |
| --- | --- | --- | --- | --- | --- |
| **Interviewees** | **Number of Participants** | ***Kebeles*** | ***Kebeles*** | **Number of Participants** | **Number of FGDs** |
| Family member | 6 | Genetua, Sendeba, Shimbrima | Addisge | 8 | 1 |
| Treatment completed | 4 | Yewish | Daget | 6 | 1 |
| HEW | 3 | Sendeba, Shimbrima, Yewish | Enaskay | 7 | 1 |
| CPA Supervisor | 2 | Debre Markos | Gudalema | 8 | 1 |
| CPA | 1 | Yewish | Mislawash | 8 | 1 |
| Patient | 1 | Shimbrima | Jama | 8 | 1 |
| WoHO Officer | 1 | Amber Zuria | Zinkir | 8 | 1 |
| Voluntary withdrawal | 1 | Amber Zuria | Zengoba | 10 | 1 |
|  |  |  | Wonga Nifasam | 8 | 1 |
|  |  |  | CPA | 8 | 1 |
| **Total** | **19** |  |  | **79** | **10** |

**Perceived effectiveness**

The major issue for patients, their families and treatment providers was the loss of working capacity, subsequent financial loss and psychosocial problems associated with living with podoconiosis lymphoedema and acute attacks. Overwhelmingly, process evaluation participants considered the intervention to be effective in terms of improving the health, economic and social aspects of patients’ lives, particularly through impact on the frequency, duration and severity of acute attack episodes -

“Previously, I used to have *mitchader* [acute attack] day-after-day…but now, I have it may be once a month only…before, we used to lag behind on tasks such as crop gathering, but now we finish at the same time as others…why, because we’re healthy” (FGD, P010, patient, male).

“Before she started the treatment, her feet used to get puffed-up, now that’s no more…previously; she used to get ill [acute attack] everyday …she recently got ill, but she got better immediately and the swelling, has decreased very much… wearing shoes helped reduce the swelling” (IDI, P061, family member, male).

Patients said that they could now work on their farm and do other non-farm activities at the same pace as their non-affected counterparts. Importantly, many mentioned that they could now earn a living–

“We don’t sit idly like we used to do previously…now, praise to God, we’re working” (FGD, P044, patient, male).

In addition to the physical improvements, patients also commented on the changed perceptions of those around them. People who had previously faced humiliating insults from non-affected community members expressed how these attitudes and remarks had changed -

“Surely, it [the treatment] has made a difference…people now say ‘look at him jumping around as he wishes’…otherwise, they used to insult us…previously, we used to be insulted as ‘saddle feet’…now people are saying ‘this treatment has benefitted you’” (FGD, P004, patient, male).

Changed perceptions extended beyond verbal to behavioural - previously, patients felt ashamed to mix with people at social and religious events, including church, ‘*Mahiber*’ and ‘*Senbete*’ (a voluntary religious gathering to celebrate a saint’s day and feed the poor). However, patients explained that this had changed-

“Before, I was unable to go to relatives’ houses, no annual celebrations, no wedding ceremonies and no church…Now I can go as I wish” (FGD, P031, patient, female).

Focus groups with CPAs corroborated the effectiveness of the intervention in improving a range of aspects of patients’ lives, particularly in reducing incidence and duration of acute attack:

“During the monthly meetings, we go around and ask what difference they’ve had observed in the past month and most patients tell us ‘since we began the treatment, we’ve not experienced *mitchader* ‘[acute attack], or ‘the *mitchader* has reduced’” (IDI, P056, CPA supervisor, female).

Only one patient who attended Monthly Intervention Meetings for 12 months had a negative opinion of the treatment. He compared the treatment with that given at another treatment site he had apparently attended three years back. The treatment procedure there included addition of bleach to the soaking water as an antiseptic -

“There’s nothing they’ve done for me…for me, there’s nothing that had improved…Nothing! Not even change the size of a needle hole…I never got better… I used to get treatment at [name of treatment site removed] and, there’s a chemical we used that we measure with a small cup [bleach] that decreased the swelling a little” (IDI, P099, treatment completed patient, male).

A female participant who voluntarily left the study after 6 months claimed that unless some form of tablet or infection treatment was included, then the Monthly Intervention Meetings were unnecessary and the feet could be washed at home.

“I wash my feet there and come home…and, I can also wash at home…the cause is sweating and *mitch*…you don’t give us tablets to swallow…and if it’s only about washing my feet, my question is, can I not wash at home? And, I decided not to come because I can wash at home” (IDI, P095, voluntary withdrawal from study, female).

In sum, almost all patients and their families and treatment providers thought the intervention was effective and recommended it for others -

“Of course the treatment has brought change…when we look at other people, even the moss is still there, and their *mitchader* [acute attack] has not decreased…we’re so much better now and able to come and go as we wish…and, if they get the same treatment, their condition will definitely improve” (FGD, P084, patient, male).

Participants were asked to describe which components of the intervention package they thought brought the changes they observed. Most indicated that it was the combined effect of all the components.

“Everything together helped: washing and the ointment and wearing shoes “(FGD, P017, patient, female).

A subtle prioritization of some parts of the package came through the Health Extension Worker interviews -

“Wearing shoes is most useful…because, if we perform the other procedures but don’t wear shoes, it will be meaningless…washing with soap, doing the exercise and applying the ointment each have their own benefits…in general, they’ve to do everything in the package” (IDI, P076, HEW, female).

**Fidelity – understanding of and adherence to the foot care and hygiene intervention**

Participants said that most patients understood the intervention procedure and had little difficulty performing the treatment at home. They were able to describe the procedures accurately -

“First, we soak our feet, then we wash with soap and clean water, rotate our feet 10 times this way and 10 times that way and move our feet…after that we apply the ointment, put a pillow under our feet and go to bed” (FGD, P015, patient, female).

However, CPAs injected an element of reality, and explained that although most patients reported performing the treatment at home every day, some did not manage this because of illness or fatigue after a long day’s work on the farm -

“Some people, as they get very tired after a long day’s work…if you ask them in a friendly way, they’ll tell you the truth…they say ‘as I’ll be very tired in the evening, I may sleep without washing my feet’…and others say ‘as soon as I get home from work, I wash my feet before I sleep’…so we can’t say every patient will follow the procedure one hundred percent” (FGD, P048, CPA, female).

Patients and CPAs agreed that those who didn’t perform the treatment procedure on a daily basis were the ones who felt the treatment had made little difference to their condition -

“There’re some people who say they’re are not getting better, the reason being, they’re not following the treatment properly. I’m getting better, except for a couple of episodes [acute attacks] recently, I’ve never been ill…we’ve benefitted from the treatment” (FGD, P035, patient, male).

**Feasibility**

Discussion of patients who were not able to perform self-treatment led to broader exploration of the feasibility of the intervention. Several patients thought that the attitude and approach of the patient was important -

“The procedure needs a very strong-willed person who can take care of himself everyday…therefore, the treatment is difficult for someone who easily gets bored” (FGD, P030, patient, male).

Others described practical barriers to treatment for certain groups of patients, including the elderly or those most disabled by the condition, especially in relation to fetching water.

“If these people can’t get support, it’d be difficult…even if they can get enough water for drinking, getting additional water for washing their feet every day would be difficult …the solution therefore would be to find someone to help them until they get better…for those who have children, their children should be encouraged to help” (FGD, P035, patient, male).

Almost all family members who participated explained the ways in which they helped, for example, by fetching water and washing the patients’ feet -

“I give him medications like Aspirin and *hareg resa* [a local herb that is boiled generating steam that is inhaled by patients believed to have mitchader] when his *mitchader* [acute attack] starts…when his basin is broken I buy him a new one…me and the other children fetch him water…when he gets ill, we wash his feet” (IDI, P063, family member, female).

The issue of water for the intervention was brought up in all interviews and focus groups when feasibility was being discussed. In most of the study *kebeles,* availability of water was not a concern. However, in some, either the distance to the water source was considerable, or water was scarce. Even if there were places nearby where underground water existed, digging wells was in places prohibited by law. A very few patients reported that community members prevented them from fetching water for treatment from communal sources.

“Since people say ‘you’re going to dry out the *kebele’s* water source’…I fetch water with Jerry cans from the spring” (FGD, P028, patient, male).

Clearly, water access is vital to treatment, and patients who are less able to walk and carry water may be at particular disadvantage if water is not freely available nearby. However, when asked about continuing treatment in the future, participants did not mention water access as a key barrier.

**Satisfaction with delivery of the intervention**

Almost all participants were satisfied with the way the intervention was delivered. These included factors such as distance from the intervention sites, materials provided and interaction with the study team. Patients mentioned travelling from 10 minutes to one and a half hours to reach the sites. Both patients and CPAs agreed that the interaction between them over the time of the intervention was very good. Several patients made complimentary remarks about the attitude and approach of the CPAs -

“Ah, their [CPAs] conduct! Even when some people come with their feet unwashed, they politely ask the reason, and politely teach them how to do the treatment the right way” (FGD, P028, patient, male).

Respondents were also asked about the supplies they were provided with. Most patients said that the soap and Whitfield ointment were sufficient. However, responses around the shoes provided were less sanguine, particularly from patients: most did not like the leather type provided. First, they were too heavy for long distance walking – canvas alternatives were suggested. Secondly, they did not reach above the ankle, so were not suitable for farming activities since soil entered and got trapped inside. Thirdly, both male and female participants said that community members thought the shoes were designed for men. Thus, to avoid ridicule, women would not wear them at church, weddings or other community functions. Finally, several patients thought the shoes made the feet seem more swollen and marked them out as having podoconiosis. All these reasons contributed to dissatisfaction with the shoes provided.

“One problem we have with the shoes is when we wear them on the farm, soil comes into the shoes…on the other hand, men have experiences of wearing shoes and people won’t say anything…however, they laugh and make jokes about women saying they’re wearing men’s shoes…thus, women don’t wear it. So it’s better if the model is designed for women” (FGD, P010, patient, male).

**Sustainability**

Patients were asked whether they would continue taking care of their feet on their own in the future, what would hinder them from continuing and their suggestions for what might be done. The main barriers they identified included affordability of the intervention products, Whitfield’s ointment, soap, shoes and socks. Some said they would do whatever it took, but others were not sure they would continue -

“Through you, we got our feet back…those of us who’ve the means, will continue the treatment…however, if it’s beyond our means, we’ll have to return to our previous condition of despair and illness…if we couldn’t afford shoes and soaps, our feet will definitely be dirty” (FGD, P084, patient, male).

“Since we’re told the treatment will be stopped…and since we’ve seen the benefits, we’ll struggle to continue…but, as life is very wicked, we may be forced to buy salt instead of soap…so we can’t say we’ll be able to continue the treatment the way we’ve been doing here so far” (FGD, P080, patient, male).

A *woreda* Health Office expert outlined the difficulties patients would face in purchasing supplies -

“You know what, compared to others, these patients are not productive, it is not that they couldn’t work, it’s simply that they spend much of their time caring for their illness than working…and when you look at it in economic terms, when they say ‘if treatment is stopped we’ll also stop’, they’re taking their assets into consideration…because, they can’t afford the treatment supplies, they ask it to be transferred to the health insurance scheme” (IDI, P066, WoHO, expert, male).

A CPA supervisor, on the other hand, suggested that not being able to afford treatment products arose from lack of understanding -

“People spend money on many things including for making festivities several times a year…how can shoes be something unaffordable? When you go to rural areas, most people go barefoot…however; the reason for it is not due to lack of money…but, buying shoes out of their pockets, is something unacceptable, that’s their problem…they’d rather make a festivity of some kind and invite people…I don’t think its lack of money alone but also of ignorance and lack of awareness” (IDI, P056, CPA supervisor, female).

These two opinions illustrate the ends of a spectrum concerning patients’ autonomy, one highlighting the need for structural changes to ensure provision for this disadvantaged group, the other perceiving information as key to empowerment and self-determinism.

Finally, in discussions regarding ways to improve the treatment in the future, the addition of tablets and injections for the treatment of acute attacks were suggested –

“The treatment itself is only washing, doesn’t have tablets or injections…it’d have been better if these were included in the treatment” (IDI, P096, patient, male).

“The rest is getting better, although not completely gone…we get the ‘*girifat’* [mild form of acute attack] if we’re struck by the sun…so give us injections or tablets” (FGD, P018, patient, female).

These are appropriate suggestions, in that a complete care programme should include treatment for as well as prevention of acute attacks. Treatment would take the form of antibiotics and antipyretic/analgesic agents.

**Discussion**

The quantitative findings demonstrated reduced incidence of acute attacks (incidence was 19.4 (95% CI 18.9 to 19.9) and 23.9 (95% CI 23.4 to 24.4) episodes per person year in the intervention and control groups respectively; incidence rate ratio 0.81 (95% CI 0.69 to 0.96, p=0.02), rate difference -4.5 (95% CI -5.1 to -3.8) episodes per person year)[[17](#_ENREF_17)]. The intervention was therefore ‘successful’, demonstrating that under pragmatic field circumstances, the foot hygiene intervention reduced acute attacks, the main clinical consequence of leg swelling. In this situation, the most important role of the process evaluation is to understand why, and how it can be optimized[[21](#_ENREF_21)]. Results of this process evaluation were consistent, establishing that from the participants’ perspective, the intervention helped reduce the frequency, duration and severity of acute attacks. This finding is also consistent with observational studies which have reported effectiveness of lymphoedema management in reducing acute attacks among patients with LF [[31-34](#_ENREF_31)]. Patients also stated that the intervention improved their social and working lives, which is consistent with studies of patients with LF[[30](#_ENREF_30), [31](#_ENREF_31), [35](#_ENREF_35)].

This process evaluation demonstrated that the hygiene and foot care intervention was considered to be feasible in daily life with minimal side effects. This is broadly consistent with the quantitative findings on adherence, which was high for washing legs with soap, applying ointment, bandaging, elevation and exercise (adherence >85% weekly) for the twelve month period[[17](#_ENREF_17)].

Fidelity was also high in terms of patient understanding and recollection of instructions given about the intervention. Fidelity in regard to ability to perform the intervention at home depended on access to supplies and water. Shortage of water and affordability of soap, ointment and shoes were also mentioned as potentially hindering patients from practicing the intervention as prescribed in the future. Inappropriate design of shoes has been reported to be a barrier to shoe use previously in northern Ethiopia[[27](#_ENREF_27)].

Shortage of water was not universally considered a problem by the participants. However several patients commented that water access was difficult for older and more disabled patients, and a few had met specific local barriers. Family members explained their role in fetching water for patients experiencing difficulties. The importance of water, sanitation, and hygiene (WASH) is increasingly recognized as essential for the control and elimination of several Neglected Tropical Diseases (NTDs), including trachoma, soil-transmitted helminths infection, and schistosomiasis[[36](#_ENREF_36)]. In a one-year follow up study of the effectiveness of lymphoedema treatment in southern Ethiopia, shortage of water, especially during the dry season, was also mentioned to be a limiting factor for self-treatment [[7](#_ENREF_7)]. Plans to scale up this intervention in other contexts should include discussion to identify and remove barriers to water access for podoconiosis patients, or adopt innovations such as the use of glycerol in smaller volumes of water[[37](#_ENREF_37)].

Support to sustain daily self-care including improved access to supplies such as soap, topical antibacterial and antifungal agents, and oral antibiotics may be challenges that need to be considered[[24](#_ENREF_24)]. The *Woreda* Health Officer referred to including these supplies within state health provision. This proposition is a point of active discussion within the Ministry of Health currently. Previous studies have found that "remoteness from a clinic site" and recurrent episodes of acute attacks are important reasons for clinic non-attendance and have suggested future lymphoedema treatment should be incorporated into existing government health care facilities in rural areas. Our participants said that experiencing acute attacks was the main reason for non-attendance at monthly meetings, consistent with findings from northern and southern Ethiopia[[25](#_ENREF_25), [26](#_ENREF_26)].

The GoLBeT RCT was sited in a high prevalence setting in northern Ethiopia, and questions about its generalizability are valid. These are discussed at length in another article[[18](#_ENREF_18)], but are worth summarizing here. We aimed to preserve high internal validity even though the RCT was intentionally pragmatic, to replicate as far as possible the field circumstances under which care provision will be offered in future. As a disease of poverty which occurs where people do not have access to or cannot afford shoes, the communities affected globally tend to be remote and impoverished, as evidenced by recent studies in Cameroon and Uganda[[38](#_ENREF_38), [39](#_ENREF_39)], often with limited water access. While the exact structures of the health system will vary from setting to setting, the barriers faced by patients (distances, water access) tend to be common across settings. Therefore, we consider that the results of GoLBeT are generalizable to other parts of the tropical highlands where podoconiosis is endemic.

Regarding models of future treatment provision, a study in North-Eastern Nigeria compared three models of delivery of lymphoedema care; Community Care: where a community member would be selected, be trained in patient care and be responsible at community level; Patient Care: where a leader of a group of patients received skills training and was responsible for training and clinical management of other members; and Health Center: where health centers were strengthened for lymphoedema management, and health personnel in charge demonstrated management at home. The findings indicated that Community Care was the most effective strategy in this setting[[35](#_ENREF_35)]. Although not identical, this model was similar to that adopted in GoLBeT, with CPAs trained to be responsible for community-level lymphoedema care. However, CPAs require an onward referral system for the prescription of antibiotics and analgesics for acute attacks and for patients with complicated lymphoedema.

As for LF management, opportunities for integration of podoconiosis management with other chronic health care programs such as leprosy and diabetes must be explored[[40](#_ENREF_40)], together with evaluation of the relative benefits of provision of care through non-government organisations compared to integration into state health systems[[41](#_ENREF_41)].

While this process evaluation had the benefits of being run in parallel with the RCT, good geographic representation and inclusion of the views of a wide range of stakeholders, it was subject to several limitations. Most important of these was that we only included one patient who had withdrawn from the RCT, despite attempts to interview more who had withdrawn voluntarily. This may have biased responses towards more favourable opinions on the intervention and its effects. Secondly, data were analysed in English rather than in Amharic, the language they were acquired in. This was to make the use of NVivo software possible, and because publication in an English language journal was anticipated. This may have led to slightly reduced richness and loss of meaning [[42](#_ENREF_42)].

This process evaluation supports the RCT’s main quantitative results[[17](#_ENREF_17)], indicating that the foot care and hygiene intervention brings about health, social and economic improvements to patients’ lives. The intervention was understood and correctly practiced by most patients and was considered feasible by most participants. Although most patients indicated they would make efforts to continue performing the intervention in the future, several questions around sustainability were raised. Future treatment implementation in other contexts should take into account factors affecting fidelity and sustainability including availability of adequate water and design of high quality footwear appropriate for women as well as men.

**Declarations**

**Ethical Approval and Consent to Participate**

Ethical approval for the study was obtained from the Ethics Review Committee of the Amhara Regional Health Bureau (reference number HRTT/11/127/08; approval date: 22/01/2016). While participants in the RCT had given written informed consent, participants in the process evaluation gave oral consent to participate, which was recorded.

**Consent for Publication:** Not applicable.

**Availability of supporting data:** The datasets used and/or analysed during the current study are available from the corresponding author on reasonable request.

**Competing Interests:** The authors declare they have no competing interests.

**Funding:** GoLBeT was jointly funded by the UK Medical Research Council, the Wellcome Trust and UKAID, Project grant MR/K007211/1. HN was supported by a University of Sussex Chancellor’s International Studentship.

**Author contributions:** HN conceived the study, HN & GD designed the study, AN conducted the interviews, HN & AN analyzed and interpreted the data, GD reviewed major conclusions reached, HN drafted the manuscript, AN, MM & GD revised the manuscript. All authors (except HN, deceased) read and approved the final manuscript.

**Acknowledgements**: We are very grateful for logistic management provided by International Orthodox Christian Charities, Ethiopia, and for the assistance of officials at the Woreda Health Office in Amber with approaching patients in Aneded woreda.

**References**

1. Price E: **The association of endemic elephantiasis of the lower legs in East Africa with soil derived from volcanic rocks.** *Transactions of the Royal Society of Tropical Medicine & Hygiene* 1976, **4**:288-295.

2. Yakob B, Deribe K, Davey G: **High levels of misconceptions and stigma in a community highly endemic for podoconiosis in southern Ethiopia.** *Transactions of the Royal Society of Tropical Medicine & Hygiene* 2008, **102**:439.

3. Tora A, Davey, G., Tadele, G.: **A Qualitative Study on Stigma and Coping Strategies of Patients with Podoconiosis in Wolaita Zone, Southern Ethiopia.** *International Health* 2011, **3**(3):176-181.

4. Wanji S, Tendongfor N, Esum M, Che J, Mand S, Mbi CT, Enyong P, Hoerauf A: **Elephantiasis of non-filarial origin (podoconiosis) in the highlands of north-western Cameroon**. *Annals of Tropical Medicine & Parasitology* 2008, **102**(6):1-12.

5. Henok L, Davey G: **Validation of the Dermatology Life Quality Index among patients with podoconiosis in southern Ethiopia.** *British Journal of Dermatology* 2008, **159**:903-906.

6. Yakob B, Deribe K, Davey G: **Health professionals' attitudes and misconceptions regarding podoconiosis: potential impact on integration of care in southern Ethiopia.** *Transactions of the Royal Society of Tropical Medicine & Hygiene* 2010, **104**:42-47.

7. Sikorski C, Ashine M, Zeleke Z, Davey G: **Effectiveness of a Simple Lymphoedema Treatment Regimen in Podoconiosis Management in Southern Ethiopia: One Year Follow-Up.** *PLoS Neglected Tropical Diseases* 2010, **4**(11):e902.

8. Davey G, Burridge E: **Community-based Control of a Neglected Tropical Disease: The Mossy Foot Treatment & Prevention Association**. *PLoS Neglected Tropical Diseases* 2009, **3**(5):e424.

9. Negussie H, Molla M, Fegan G, P N, Enqueselassie F, McKay A, MJ N, Lang T, Davey G: **GoLBeT [Gojjam Lymphoedema Best practice Trial]: description of study protocol for a randomised controlled trial of effectiveness of treatment for podoconiosis (non-filarial elephantiasis)** *Trials* 2015, **16**:8.

10. Thorpe KE, Zwarenstein M, Oxman AD, Treweek S, Furberg CD, Altman DG, Tunis S, Bergel E, Harvey I, Magid DJ *et al*: **A pragmatic–explanatory continuum indicator summary (PRECIS): a tool to help trial designers**. *Canadian Medical Association Journal* 2009, **180**(10):E47-E57.

11. Campbell N, Murray E, Darbyshire J, Emery J, Farmer A, Griffiths F, Guthrie B, Lester H, Wilson P, Kinmonth A: **Designing and evluating complex interventions to improve health care**. *British Medical Journal* 2007, **334**:455-459.

12. Hawe P, Shiell A, Riley T: **Complex interventions: how "out of control" can a randomised controlled trial be?** *British Medical Journal* 2004, **328**:1561-1563.

13. Nanyonjo A, Nakirunda M, Makumbi F, Tomson G, Kallander K, in SSG: **Community acceptability and adoption of integrated community case management in Uganda**. *Am J Trop Med Hyg* 2012, **87**(5 Suppl):97-104.

14. Rapport F, Storey M, Porter A, Snooks H, Jones K, Peconi J, Sanchez A, Siebert S, Thorne K, Clement C *et al*: **Qualitative research within trials: developing a standard operating procedure for a clinical trials unit.** *Trials* 2013, **14**:1-8.

15. Oakley A, Strange V, Bonell C, Allen E, Stephenson J: **Process evaluation in randomised controlled trials of complex interventions.** *British Medical Journal* 2006, **332**:413-416.

16. Negussie H, Addissie A, Addissie T, Davey G: **Preparing for and Executing a Randomised Controlled Trial of Podoconiosis Treatment in Northern Ethiopia: the Utility of Rapid Ethical Assessment.** . *PLoS Neglected Tropical Diseases* 2016, **10**(3):e0004531.

17. Negussie H, Molla M, Ngari M, Berkley J, Kivaya E, Njuguna P, Fegan G, Tamiru A, Kelemework A, Lang T *et al*: **Lymphoedema management to prevent acute dermatolymphangioadenitis in podoconiosis (GoLBeT): a pragmatic randomised controlled trial in northern Ethiopia.** *The Lancet Global Health* 2018, **6**:e795-803.

18. Molla M, Negussie H, Ngari M, Kivaya E, P N, Enqueselassie F, Berkley J, Davey G: **Pragmatism in practice: lessons learned during screening and enrollment for a randomised controlled trial in rural northern Ethiopia.** *BMC Medical Research Methodology* 2018, **18**:26.

19. Central Statistical Agency: **Population and Houseing Census Report. CSA Addis Ababa Ethiopia, 2007.** .

20. Moore GF, Audrey S, Barker M, Bond L, Bonell C, Hardeman W, Moore L, O’Cathain A, Tinati T, Wight D *et al*: **Process evaluation of complex interventions: Medical Research Council guidance**. *BMJ : British Medical Journal* 2015, **350**.

21. Craig P, Dieppe P, Macintyre S, Michie S, Nazareth I, Petticrew M: **Developing and evaluating complex interventions: the new Medical Research Council guidance**. *BMJ* 2008, **337**.

22. Hsieh H, Shannon S: **Three Approaches to Qualitative Content Analysis**. *Qualitative Health Research* 2005, **15**(9):1277-1288.

23. Moore G, Audrey S, Barker M, Bond L, Bonell C, Hardeman W, Moore L, O'Cathain A, Tinati T, Wight D *et al*: **Process evaluation of complex interventions: UK Medical Research Council (MRC) guidance.** *British Medical Journal* 2015, **350**:h1258.

24. Tsegay G, Wubie M, Degu G, Tamiru A, Cooper M, Davey G: **Barriers to access and re-attendance for treatment of podoconiosis: a qualitative study in northern Ethiopia**. *Int Health* 2015, **7**(4):285-292.

25. Campion A, Tamiru A, Tsegay G, Davey G: **Reasons for loss to follow-up of patients with podoconiosis in the Amhara Region, northern Ethiopia**. *Int Health* 2015, **7**(5):367-373.

26. Tora A, Davey G, Tadele G: **Factors related to discontinued clinic attendance by patients with podoconiosis in southern ethiopia: a qualitative study.** *BMC Public Health* 2012, **12**:902.

27. Kelemwork A, Tora A, Amberbir T, Agadaw G, Asmamaw A, Deribe K, Davey G: **‘Why should I worry, since I have healthy feet?’ A qualitative study exploring barriers to use of footwear among rural community members in Northern Ethiopia**. *BMJ Open* 2016, **6**:e010354.

28. Addiss DG, Louis-Charles, J., Roberts, J., LeConte, F., Wendt, J. M., Milord, M. D., Lammie, P. J., Dreyer, G.: **Feasibility and Effectiveness of Basic Lymphedema Management in Leogane, Haiti, an Area Endemic for Bancroftian Filariasis.** *PLoS Neglected Tropical Diseases* 2010, **4**(4):e668.

29. Jullien P, Some J, Brantus P, Bougma R, Bamba I, Kyelem D: **Efficacy of home-based lymphoedema management in reducing acute attacks in subjects with lymphatic filariasis in Burkina Faso.** *Acta Tropica* 2011, **120S**:S55-S61.

30. Budge P, Little K, Mues K, Kennedy E, Prakash A, Rout J, Fox L: **Impact of Community-Based Lymphedema Management on Perceived Disability among Patients with Lymphatic Filariasis in Orissa State, India**. *PLoS Neglected Tropical Diseases* 2013, **7**(3):e2100.

31. Suma TK, Shenoy RK, Kumaraswami V: **Efficacy and sustainability of a footcare programme in preventing acute attacks of adenolymphangitis in Brugian filariasis**. *Tropical Medicine and International Health* 2002, **7**(9):3

32. Addiss DG, Louis-Charles J, Roberts J, LeConte F, Wendt JM, Milord MD, Lammie PJ, Dreyer4 G: **Feasibility and Effectiveness of Basic Lymphedema Management in Leogane, Haiti, an Area Endemic for Bancroftian Filariasis**. *PLoS Negl Trop Dis* 2010, **4**(4):1-8.

33. Jullien P, Soméb JdA, Brantusc P, Bougmad RW, Bambad I, Kyelemd D: **Efficacy of home-based lymphoedema management in reducing acute attacks in subjects with lymphatic filariasis in Burkina Faso**. *Acta Tropica* 2011, **120s**:S55-S61.

34. Budge PJ, Little KM, Mues KE, Kennedy ED, Prakash A, Rout J, Fox LM: **Impact of Community-Based Lymphedema Management on Perceived Disability among Patients with Lymphatic Filariasis in Orissa State, India**. *PLoS Negl Trop Dis* 2013, **7**(3):1-11.

35. Akugn OB, Badakib JA: **Management of adenolymphangitis and lymphoedema due to lymphatic filariasis in resource-limited North-eastern Nigeria**. *Elsevier* 2010 **Acta Tropica 120S**: S69-S75.

36. Stocks ME, Ogden S, Haddad D, Addiss DG, McGuire C, Freeman MC: **Effect of water, sanitation, and hygiene on the prevention of trachoma: a systematic review and meta-analysis**. *PLoS Med* 2014, **11**(2):e1001605.

37. Brooks J, Ersser S, Cowdell F, Gardiner E, Mengistu A, Matts P: **A Randomised Controlled Trial to evaluate the effect of a new skin care regimen on skin barrier function in those with podoconiosis in Ethiopia.** . *British Journal of Dermatology* 2017.

38. Wanji S, Kengne-Ouafu J, Deribe K, Tembei A, Njouendou A, Tayong D, Sofeu-Feugaing D, Datchoua-Poutcheu F, Cano J, Giorgi E *et al*: **Study of lymphoedema of non-filarial origin in the northwest region of Cameroon: spatial distribution, profiling of cases and socio-economic aspects of podoconiosis.** *International Health* 2018.

39. Kihembo C, Masiira B, Lali W, Matwale G, Matovu J, Kaharuza F, Ario A, Nabukenya I, Makumbi I, Musenero M *et al*: **Risk Factors for Podoconiosis: Kamwenge District, Western Uganda, September 2015**. *American Journal of Tropical Medicine & Hygiene* 2017, **96**(6):1490-1496.

40. Mitjà O, Marks M, Bertran L, Kollie K, Argaw D, Fahal AH, Fitzpatrick C, Fuller LC, Garcia Izquierdo B, Hay R *et al*: **Integrated Control and Management of Neglected Tropical Skin Diseases**. *PLOS Neglected Tropical Diseases* 2017, **11**(1):e0005136.

41. Deribe K, Kebede B, Tamiru M, Mengistu B, Kebede F, Martindale S, Sime H, Mulugeta A, Kebede B, Sileshi M *et al*: **Integrated morbidity management for lymphatic filariasis and podoconiosis, Ethiopia.** *Bulletin of the World Health Organisation* 2017, **9**:652-656.

42. van Nes F, Abma T, Jonsson H: **Language differences in qualitative research: is meaing lost in translation?** *European Journal of Ageing* 2010, **7**:313-316.
